# Evaluating the Feasibility of a Nonelectric Bubble CPAP System in the NICU of a Philippine Regional Referral Hospital: A Qualitative Study

**DOI:** 10.1155/ijpe/5529978

**Published:** 2025-03-10

**Authors:** Grace Katharine Meara, Ghassan Bou Saba, Navid Roodaki, Daisy Garcia, Agustin Conde-Agudelo, Paula Rauschendorf, Thomas F. Burke

**Affiliations:** ^1^Vayu Global Health Foundation, Boston, Massachusetts, USA; ^2^Section of Neonatology, Department of Pediatrics, Ilocos Training and Regional Medical Center, San Fernando, La Union, Philippines; ^3^College of Medicine, Mariano Marcos State University, City of Batac, Ilocos Norte, Philippines; ^4^College of Medicine, University of the Northern Philippines, Vigan City, Philippines; ^5^Oxford Maternal and Perinatal Health Institute, Green Templeton College, University of Oxford, Oxford, UK; ^6^Department of Emergency Medicine, Massachusetts General Hospital, Boston, Massachusetts, USA; ^7^Department of Emergency Medicine, Harvard Medical School, Boston, Massachusetts, USA; ^8^Department of Global Health, Harvard T.H. Chan School of Public Health, Boston, Massachusetts, USA

**Keywords:** CPAP, CPAP usability, feasibility assessment, healthcare quality, healthcare worker assessment, neonatal respiratory distress, NICU integration, respiratory support

## Abstract

**Aim:** Bubble continuous positive airway pressure (bCPAP) is recommended by WHO for the treatment of neonatal respiratory distress; however, considerable challenges hinder global access. The objective of this study was to evaluate the feasibility of the use and integration of Vayu bCPAP systems into the neonatal intensive care unit of a public regional referral hospital in the Philippines.

**Methods:** We conducted a mixed-methods study from March 2021 to May 2022. Demographic and clinical characteristics of 150 consecutive neonates treated with a Vayu bCPAP system were collected and analyzed. Forty-seven healthcare workers participated in a survey, and 5-point Likert scales were used to assess the usability and integration of Vayu bCPAP systems into the hospital.

**Results:** The mean duration of bCPAP treatment was 2.5 days (range, 1.0–7.0). Of the neonates treated with a Vayu bCPAP system, 89.3% survived to discharge. Most healthcare workers reported that Vayu bCPAP systems are easy to use. The main themes included positive perceptions of usability, accessibility of the devices, and improved neonatal outcomes. There were mixed perceptions about training on the use of the device, and improvements were suggested.

**Conclusion:** Implementation of Vayu bCPAP systems at a regional referral hospital in the Philippines was feasible. Vayu bCPAP systems were perceived to be easy to use and to improve neonatal outcomes.

## 1. Introduction

Worldwide, 46% of deaths in children under 5 years of age occur in the first 28 days of life [1]. The United Nations Sustainable Development Goal 3.2 challenges the world to reduce neonatal mortality to 12 deaths per 1000 live births by the year 2030 [2, 3]. In the Philippines, neonatal deaths constitute nearly half of all deaths among children younger than 5, where respiratory distress is the most common cause [4]. Although mortality among children younger than 5 years of age has decreased over the past two decades, neonatal mortality in the Philippines has remained unchanged during the past 5 years [5].

Interventions proven to increase neonatal survival in high-income settings included appropriate use of antenatal corticosteroids, surfactant, kangaroo mother care (KMC), mechanical ventilation, and continuous positive airway pressure (CPAP) [6–8]. CPAP has been shown to be helpful in the support of neonates with respiratory distress syndrome (RDS), pneumonia, sepsis, transient tachypnea of the newborn, neonatal apnea, and neonatal asphyxia [9]. In low- and middle-income countries (LMICs), CPAP was reported to decrease mortality among premature neonates with RDS by up to 66% and to reduce the need for mechanical ventilation by up to 50% [10]. In a recent publication from a referral hospital in the Philippines, survival among neonates in respiratory distress improved by 53% after introduction of Vayu bubble continuous positive airway pressure (bCPAP) systems [11]. Although global access to CPAP has the potential to reduce neonatal mortality and the need for invasive ventilation, several barriers limit its access including high device costs, a lack of pressurized medical air and reliable electricity, a dearth of biomedical engineering support, and weak supply chains [12].

**Figure 1:**
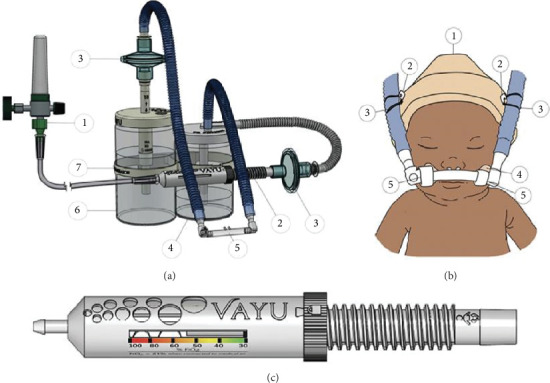
(a) The components of the bubble continuous positive airway pressure system: ① an external pressurized oxygen source, ② a Venturi blender, ③ two bacterial viral filters (one on an inspiratory and one on an expiratory limb), ④ a humidifier, ⑤ nasal prongs, ⑥ a pressure generator with an adjustable wand, and ⑦ a warmer bracket. (b) ① a hat, ② two safety pins, ③ two rubber bands, ④ a hook mustache, and ⑤ soft-loop fasteners. (c) The Vayu blender.

bCPAP has been shown to be as safe and effective as ventilator-driven CPAP [13–15]. bCPAP is characterized by the delivery of warm, humidified, and blended air and oxygen with continuous positive pressure from an adjustable expiratory column of water [16]. The Vayu bCPAP system generates continuous pressure throughout the respiratory system and delivers blended, humidified, and filtered breathing gases at precise oxygen concentrations without the need for electricity [17] (Figure 1). The Vayu bCPAP system proved feasible at Muhimbili National Hospital in Dar es Salaam, Tanzania, but the feasibility of the device in other settings needs to be further explored [18].

In March of 2021, Vayu bCPAP systems were donated to the Ilocos Training and Regional Medical Center (ITRMC) in the Philippines in response to the urgent request from the ITRMC NICU care team. The objective of this study was to assess the feasibility of use and integration of the Vayu bCPAP systems and to better understand recently published findings that introduction of these systems improved survival and decreased the need for surfactant and invasive ventilation [11].

## 2. Materials and Methods

### 2.1. Study Setting

ITRMC is a 300-bed public regional referral hospital in San Fernando City, La Union, the Philippines. The pediatric department has a Level 3 NICU with 25 beds, which can be increased to 40 beds when needed. The NICU was equipped with radiant warmers, incubators, and monitoring equipment to care for its admitted neonates. Support for mothers to breast-feed their neonates was provided 24 h daily. Surfactant therapy was available, and the cost was covered by the Philippines Health Insurance Corporation. One attending neonatologist and experienced pediatricians were readily available in-house during daylight hours, and pediatric residents were available at night. The average patient-to-nurse ratio was 10 neonates to every nurse.

CPAP therapy was initiated in neonates with respiratory distress that had respiratory severity scores (RSSs) of 5 or greater and at the discretion of the clinician's judgement. Neonates with RDS were clinically classified as mild, moderate, and severe. Radiologists also provided an RDS grade based on admission chest radiographs. CPAP was not initiated in the delivery room, and there was no practice of prophylactic CPAP. Orogastric tubes were placed in all neonates treated with CPAP. All neonates on respiratory support of any kind had continuous pulse oximetry monitoring.

### 2.2. The Intervention

Four Vayu bCPAP systems were introduced to ITRMC in early March 2021. Training on Vayu bCPAP systems was provided virtually on Zoom by the implementation team of the Vayu Global Health Foundation. The training covered assembly and application of the device, troubleshooting, and reprocessing (cleaning and disinfection). The two trainees of the initial virtual training session assumed the roles of master trainers. The master trainers trained the remaining NICU staff. Knowledge and skills were transferred via one-to-one mentorship during shifts in the NICU. The NICU staff were asked to complete an online training program [19]. Ten additional Vayu bCPAP systems were introduced in April 2021.

Over the study period, a total of 14 Vayu bCPAP systems, 2 Fisher & Paykel CPAP machines, and mechanical ventilators in CPAP mode were available for use at the discretion of the NICU staff. There was no set number of available mechanical ventilators because the NICU shared the hospital's 30 mechanical ventilators (Mindray, Puritan Bennett, and Hamilton-G5 ventilators) with all departments.

Guidelines regarding CPAP therapy decision-making did not change prior to and following the introduction of Vayu bCPAP systems. There were no changes to any care protocols before and after introduction of Vayu bCPAP systems including practices of feeding, use of antenatal steroids, administration of caffeine or surfactant, availability of antibiotics, availability of oxygen, and access to invasive ventilation.

### 2.3. Clinical Characteristics of Neonates Treated With Vayu bCPAP Systems

Clinical characteristics of all neonates treated with a Vayu bCPAP system between March 2021 and February 2022 were prospectively recorded. The following deidentified data were collected: date and time of birth, admission date, Apgar scores at 1 and 5 min, estimated gestational age, sex, birthweight, recorded diagnoses, administration of surfactant, date and time of initiation and termination of CPAP therapy, survival status, and complications. Data were collected by residents rotating in the NICU under the supervision of the ITRMC research team and uploaded securely to a shared database. Data fidelity and quality were reviewed weekly by the research teams at ITRMC and the Vayu Global Health Foundation. Standard descriptive analyses (means, medians, and ranges) of the clinical characteristics were performed with the use of Microsoft Excel software (Microsoft).

### 2.4. Healthcare Worker Survey

A survey to assess usability, durability, barriers and facilitators, assembly, monitoring, troubleshooting, cleaning, reprocessing, and recommendations was developed iteratively by the Vayu Global Health Foundation and the ITRMC's research team. The survey was administered with the use of the KoboToolbox application (Kobo Inc.) and piloted in the Philippines for cultural appropriateness and validity. The survey was conducted between April 26, 2022, and May 14, 2022. During this period, a designated room with two tablets was available for healthcare personnel to have a private and quiet space in which to complete the survey anonymously. An independent moderator was always present to provide the consent form and to ensure confidentiality. A translator was available at all times. Personal identifying information was not collected. The survey used standard numerical 5-point Likert scales to score tasks as “*very easy*,” “*easy*,” “*neutral*,” “*difficult*,” or “*very difficult*” to perform.

After survey completion, data were uploaded to a secure KoboToolbox server. Standard descriptive analyses of the numerical-scale responses were reported as means, medians, and ranges. Written responses were aggregated and analyzed in thematic clusters.

### 2.5. Ethical Considerations

The study was approved by the ethical review board of ITRMC (ITRMC REC-2022-04). Written informed consent was obtained from healthcare workers before the survey was administered. The consent form included a confidentiality clause and guarantee that the respondents' employment would not be affected by their responses.

## 3. Results

### 3.1. Neonatal Outcomes

In total, 979 neonates were admitted to the ITRMC NICU from March 1, 2021, to February 26, 2022. Of these 979 neonates, 193 (19.7%) were treated with CPAP, of which 150 (77.7%) were with a Vayu bCPAP system. Among the neonates treated with a Vayu bCPAP system, 7 (4.7%) neonates had birthweights less than 1000 g, 23 (15.3%) were between 1000 and 1499 g, 72 (48.0%) between 1500 and 2499 g, and 48 (32.0%) greater than or equal to 2500 g. Survival to discharge rates were 57.1%, 69.6%, 94.4%, and 95.8%, respectively. The mean gestational age of all neonates who received treatment with a Vayu bCPAP system was 34.6 weeks (range, 27–40 weeks). A total of 4 neonates (2.7%) were less than or equal to 28 weeks gestational age, 22 (14.7%) between 28 and 31 weeks gestational age, 61 (40.7%) between 32 and 36 weeks gestational age, and 63 (42.0%) were 37 or greater weeks gestation.

The most common diagnosis among neonates treated with a Vayu bCPAP system was RDS (47.3%). RDS diagnoses were divided into severe (67.6%), moderate (31.0%), and mild (1.4%). Of the neonates treated with a Vayu bCPAP system, 35.3% were diagnosed with pneumonia (Table 1). The mean duration of Vayu bCPAP system treatment was 2.5 days (range, 1.0–7.0 days). Of the 150 neonates treated with a Vayu bCPAP system, 134 (89.3%) survived to discharge, and 16 (10.7%) died. Of the 16 deaths, 3 (18.8%) neonates had an extremely low birthweight, 7 (43.8%) a very low birthweight, and 5 (31.3%) a low birthweight.

Among the 150 neonates treated with a Vayu bCPAP system, it served as the primary advanced (beyond low-flow oxygen therapy) respiratory intervention in 119 cases (79.3%). The Vayu bCPAP system was considered the primary advanced respiratory intervention for a neonate if it was their first advanced form of respiratory support after being admitted to the NICU. There were no reported complications associated with Vayu bCPAP systems. Of the patients placed on a Vayu bCPAP system, 15 neonates (10.0%) were escalated to a more intensive form of respiratory support (noninvasive positive pressure ventilation (NIPPV) or intubation) and 66 (44.0%) received surfactant.

### 3.2. Healthcare Worker Survey

#### 3.2.1. Survey Responses

All 47 NICU healthcare workers who were invited to participate in the study completed the survey. Thirty-seven were female, and 10 were male (Table 2). The median age was 32 years. The job designations included 24 nurses (51%), 8 pediatric residents (17%), 4 nurse attendants (9%), 3 consultant level pediatricians (6%), 3 midwives (6%), 2 neonatologists (4%), and 3 persons (4%) who did not report their job designation. Among the survey respondents, 24 (51.6%) had worked with neonates at ITRMC for at least 6 years.

Healthcare workers were asked to assess the ease of application of the nasal prongs and the breathing tubes. The respondents' mean rating for ease of setup was 3.7 on a 5-point Likert scale, with higher values indicating greater ease. The mean time required to apply a Vayu bCPAP system was 9 min. When asked about how easy it was to identify proper functioning of a Vayu bCPAP system during treatment, the respondents' mean score was 4.1. None of the participants responded “*difficult*” or “*very difficult*.” When asked about the ease of troubleshooting to address malfunctions, respondents reported a mean score of 4.0. None of the respondents indicated that it was difficult or very difficult to identify and fix a problem with a Vayu bCPAP system. The mean score was 4.0 for ease of cleaning and reprocessing of the Vayu bCPAP system and its associated components. None of the participants responded that it was very difficult to clean and reprocess the system.

Overall, 92% of the respondents were “*very satisfied*” (60%) or “*satisfied*” (32%) with the Vayu bCPAP systems. No participants responded that they were unsatisfied or very unsatisfied with the Vayu bCPAP system. When asked how likely they were to recommend the Vayu bCPAP system to other healthcare workers and facilities, 38 healthcare workers (81%) responded “*very likely*” and 8 (17%) responded “*likely*.” No respondents indicated that they were unlikely or very unlikely to recommend the Vayu bCPAP system. Results of the healthcare worker surveys are summarized in Table 3.

#### 3.2.2. Open-Response Questions

A total of 19 participants (40%) provided comments on the Vayu bCPAP system. The main themes in the open responses included perceptions on usability and accessibility of the device, perceptions on neonatal outcomes, and perceptions on training (Table 4). The availability of other CPAP devices at ITRMC also emerged as a main theme.

##### 3.2.2.1. Positive Perceptions of Device Usability

When asked about the Vayu bCPAP system, respondents frequently commented on device usability, including device assembly, operation, and the ability to identify a malfunctioning device. The most common sentiment reported was that the Vayu bCPAP system was easy to assemble. When participants were asked what device they would recommend for use in other facilities, one participant stated: “I recommend the Vayu bCPAP [more] than the other types of CPAP. It is easier to set up” (Participant 2). A participant highlighted that Vayu bCPAP systems were useful in time-critical situations (Participant 8).

Participants also noted that the devices were easy to operate. One participant said, “I would recommend Vayu CPAP since it is very handy and easy to use” (Participant 10). Another survey respondent recommended Vayu bCPAP systems for lower-level facilities because of their simplicity (Participant 32).

Respondents indicated that it was straightforward to identify whether a Vayu bCPAP system was malfunctioning. One participant stated that it was easier to identify problems with a Vayu bCPAP system than with other CPAP devices: “With Vayu CPAP, it is easier to tell if something is not functioning properly” (Participant 16). Participants also highlighted that the Vayu bCPAP system did not require advanced bioengineering skills to use on patients (Participants 34 and 15).

##### 3.2.2.2. Negative Perceptions of Device Usability

Some participants had mixed feelings regarding the usability of the Vayu bCPAP system. One respondent reported that although the devices were beneficial, they were difficult to set up: “Though it is hard to set up, the benefit of this device is very much commendable” (Participant 18). A survey respondent noted that it can take time to assemble the Vayu bCPAP system, but it works very well as a treatment option in the ITRMC NICU (Participant 20).

##### 3.2.2.3. Perceptions of Accessibility

Most participants reported that Vayu bCPAP systems have features that make them ideal for resource-constrained settings. One participant stated: “I would recommend the Vayu CPAP since… it can be used in resource-limited settings like the Philippines” (Participant 10). Another participant noted that the device could be helpful in various settings: “[I recommend] Vayu CPAP since it can be used in remote areas where there is limited equipment” (Participant 43).

Respondents recommended Vayu bCPAP systems because of affordability and lack of a requirement for electricity or compressed air. One participant noted: “I will recommend Vayu CPAP to other facilities because it is a low cost-effective device compared to other devices” (Participant 25). Another respondent recommended Vayu bCPAP systems because “Vayu is cheaper and does not require compressed air” (Participant 44).

##### 3.2.2.4. Perceptions of Neonatal Outcomes

Many participants reported that they perceived an improvement in neonatal outcomes since the implementation of Vayu bCPAP systems. One participant noted: “Since we started using Vayu CPAP in our institution, there was a noted decrease in the chance of a patient being intubated” (Participant 18). Another participant recommended Vayu bCPAP systems for other facilities because they similarly perceived a decrease in intubation: “I recommend Vayu CPAP because it is a low-cost device that allows delivery of oxygen flow, concentration, and pressure that can improve the outcomes of patients and lessen the patients from intubation” (Participant 6). One participant shared: “Vayu CPAP is very useful to all neonates. I could say that this is an essential device in the ICU, like the mechanical ventilator, wherein many patients may recover from their critical conditions. I just hope all hospitals are capable of having more devices like this” (Participant 24).

##### 3.2.2.5. Perceptions of Device Training

Participants were asked about their training experience with Vayu bCPAP systems and how their experience could be improved. Of the 35 participants who responded, 18 (51%) provided suggestions on how to future train on Vayu bCPAP systems. The most common request was for hands-on training (33%). Other suggestions included incorporating refresher training into the hospital's annual schedule.

##### 3.2.2.6. Commercially Available CPAP Landscape

Survey respondents described the landscape of commercially available CPAP devices in the ITRMC NICU. Participants reported that the most common options for CPAP, prior to the introduction of Vayu bCPAP systems, were ventilator-driven CPAP devices but that there were barriers to their use. These barriers included the need for skilled bioengineers, high cost (ventilators have to be rented by the NICU), and the lack of availability.

When asked which CPAP system would be most challenging to incorporate into healthcare settings, one respondent stated: “The ventilator CPAP… it requires knowledgeable personnel to set up the machine” (Participant 7). Cost was a common obstacle noted regarding the ventilator-driven CPAP in ITRMC. One participant stated, “Ventilator-driven CPAP is the most challenging to incorporate into healthcare settings especially into facilities with limited resources… not all healthcare facilities can afford mechanical ventilators” (Participant 19).

Most respondents raised the issue of availability of the Vayu bCPAP systems in their NICU. One participant stated: “The Vayu CPAP is readily available in an emergency setting when there is no mechanical ventilator” (Participant 10). Another participant commented on the limited availability of ventilator-driven CPAP at ITRMC: “Availability of the ventilator right away is the main problem hence Vayu CPAP is more available in our NICU setting” (Participant 30).

## 4. Discussion

The most common themes in the survey responses were positive and negative perceptions on the usability of Vayu bCPAP systems, accessibility, the perceived effect on neonatal outcomes, the commercially available CPAP landscape, and device training. Vayu bCPAP systems were found to be easy to assemble, operate, and monitor. Additionally, neonatal survival rates were perceived to improve.

Vayu bCPAP systems and other bCPAP devices have been shown effective and safe for treatment of neonates in respiratory distress [10, 11]. In the companion quantitative study, the survival rates after the introduction of Vayu bCPAP systems at ITRMC were similar to those in other studies evaluating the survival of neonates treated with bCPAP devices in LMICs [20–22]. Therefore, increased access to affordable and appropriate bCPAP devices in newborn units around the world is imperative to reducing neonatal mortality.

The ease of use of the Vayu bCPAP system was attributed by survey respondents to the simplicity of assembly and application as well as device monitoring. These attributes facilitated integration of Vayu bCPAP systems into routine care at ITRMC. Prior to implementation of the Vayu bCPAP systems into ITRMC NICU, the main method to provide CPAP was through mechanical ventilator–driven CPAP. However, the availability of ventilators was limited, especially during the COVID-19 pandemic. Moreover, the use of ventilator-driven CPAP was limited because of the lack of consumables, the need for uninterrupted electricity, and the need for advanced technical skills.

In a recent survey conducted at 103 public and private hospitals in the Philippines, 50% of facilities reported that all their ventilators are “*often/almost always*” in use at any one time [23]. Therefore, it is no surprise that access to quality CPAP is an enormous gap and that invasive ventilation is overused. Increasing access to bCPAP with the introduction of Vayu bCPAP systems into the NICU at ITRMC freed up mechanical ventilators for patients who needed them most. Healthcare workers' perceptions that mortality and intubation rates decreased after introduction of Vayu bCPAP systems were consistent with the findings in the published quantitative companion paper [11].

Several studies have assessed the feasibility of low-cost bCPAP systems in LMICs [10, 12, 24, 25]. Various factors have been shown to be critical for optimizing outcomes, including simplicity of use, affordability, low maintenance, appropriate treatment initiation and monitoring, and availability of consumables [24]. Participants in this study noted that the Vayu bCPAP system was well positioned to overcome each of these barriers.

Some participants reported difficulties with device assembly; this may have been a limitation of the training they received. The negative perceptions of assembly of the Vayu bCPAP system were further explored. It was reported that after cleaning and reprocessing, individual components of the Vayu bCPAP system were stored separately. Therefore, when a healthcare worker identified the need to initiate the use of Vayu bCPAP treatment in a neonate, they assembled the device at that moment. This created delays in treatment initiation, especially in comparison to mechanical ventilators that by design are preassembled. For optimal use, it is now recommended that Vayu bCPAP systems are assembled after reprocessing and stored so they can be ready for immediate use.

When asked to provide feedback, over half of the survey respondents provided suggestions on training improvements. Respondents frequently recommended refresher training that included hands-on simulation. The initial instruction for trainers on Vayu bCPAP systems was entirely virtual because of the COVID-19 pandemic. Thereafter, the initial trainees conducted in-person training for other NICU staff. Only 12 of the 47 neonatal providers completed the online certificate training program; therefore, this is now mandatory for all clinicians who provide treatment using Vayu bCPAP systems [19]. A holistic bCPAP package that is nested into the local system of neonatal care may lead to improved integration and effectiveness [26, 27].

The strengths of this study are the rigorous methods used to explore the feasibility of use of the Vayu bCPAP system at ITRMC and the 100% survey participation rate (100%). There are several potential limitations. Since mechanical ventilators were often not available in the NICU of ITRMC, some neonates who met criteria for mechanical ventilation were treated with bCPAP devices instead of invasive ventilation. This practice may have negatively affected the percentage of infants who survived after being placed on bCPAP. Secondly, the use of a survey instrument to explore the perceptions of healthcare workers might have imposed a social desirability bias, a recall bias, or both. Given that the survey was conducted more than 12 months after the introduction of the Vayu bCPAP system, the participants may have had difficulty accurately recalling their experiences and perceptions regarding device training or protocols prior to device introduction. However, the 12-month interval allowed time for the intervention to settle in as a new norm for delivered care. In an effort to eliminate this risk, respondents were informed before the survey that their answers were anonymous and that their participation in the study would not affect their employment. In addition, an independent moderator was present to facilitate survey administration. Third, because of the nature of surveys, some of the themes around feasibility could have been missed. In-person interviews may have allowed for further exploration. Advantages of the survey utilized in this study, however, include the possibility of a larger sample and the clear quantification of responses with Likert scales. Furthermore, the open-ended comment section offered a space for respondents to provide greater details on their answers. Finally, the implementation period of Vayu bCPAP systems coincided with the initial months of the COVID-19 pandemic. The pandemic caused major staff shortages, rerouting of some babies with COVID-19-positive mothers to an isolation unit, and limited contact time between neonates and healthcare workers. The changes in clinical care could have limited the generalizability of the findings and created a challenging environment for training and implementation.

Future research should investigate the feasibility of using Vayu bCPAP systems in different settings such as in transport, labor and delivery, and lower-level facilities. Identifying where and how the Vayu bCPAP system can be most impactful is critical to optimizing its effect on neonatal survival.

## 5. Conclusion

It was feasible to implement Vayu bCPAP systems at a regional referral hospital in the Philippines. This system was found to be easy to use and perceived to improve neonatal outcomes.

## Figures and Tables

**Table 1:** Clinical characteristics and outcomes of neonates treated with the Vayu bCPAP system.

**Characteristics and outcomes**	**Patients (** **N** = 150**)**
Sex, no. (%)	
Male	78 (52.0)
Female	72 (48.0)
Birthweight	
Mean (range) (g)	2048 (600–4400)
Extremely low birthweight ≤ 1000 g, no. (%)	7 (4.7%)
Very low birthweight 1000–1499 g, no. (%)	23 (15.4%)
Low birthweight 1500–1499 g, no. (%)	72 (48.0%)
Normal birthweight ≥ 2500 g, no. (%)	48 (32.0%)
Gestational age	
Mean (range) (weeks)	34.6 (27.0–40.0)
≤ 28 weeks, no. (%)	4 (2.7%)
> 28 and < 32 weeks, no. (%)	22 (14.7%)
> 32 and < 37 weeks, no. (%)	61 (40.7%)
≥ 37 weeks, no. (%)	63 (42.0%)
Reported diagnosis, no. (%)	
RDS	71 (47.3)
Severe RDS	48 (32.0)
Moderate RDS	22 (14.7)
Mild RDS	1 (0.7)
Neonatal pneumonia, including meconium aspiration pneumonia	53 (35.3)
Neonatal sepsis and meningitis	14 (9.3)
Others	12 (8.0)
Duration of treatment with Vayu bCPAP system	
Mean (range) (days)	2.5 (1.0–7.0)
Outcomes, no. (%)	
Survived to discharge	134 (89.3)
Died	16 (10.7)

Abbreviations: bCPAP, bubble continuous positive airway pressure; RDS, respiratory distress syndrome.

**Table 2:** Sociodemographic characteristics of healthcare workers surveyed.

**Characteristic**	**Healthcare workers (** **N** = 47**)**	**%**
Sex		
Female	37	78.7
Male	10	21.3
Job designation		
Nurse	24	51.0
Pediatric resident	8	17.0
Nurse attendant	4	8.5
Pediatrician	3	6.4
Midwife	3	6.4
Neonatologist	2	4.3
No response provided	3	6.4
Educational attainment		
Bachelor's degree	28	59.6
Medical degree (MD, MBBS, MBChB)	13	27.6
Diploma in midwifery	6	12.8
Duration of work at ITRMC in years		
0–5	21	44.6
6–10	17	36.2
11–15	3	6.4
> 15	6	12.8
Duration of work with neonates in years		
0–2	16	34.0
3–5	7	14.9
6–10	17	36.2
11–15	4	8.5
> 15	3	6.4

Abbreviation: ITRMC, Ilocos Training and Regional Medical Center.

**Table 3:** Healthcare worker survey response distributions and averages.

**Survey questions**	**Scale used**	**Participant response**						**Average response (mean)**
		**5**	**4**	**3**	**2**	**1**	**0 (not applicable)**	
How easy or difficult is it to use this CPAP in a baby?	Very easy (5) Easy (4) Neutral (3) Difficult (2) Very difficult (1)	14 (30%)	12 (26%)	14 (30%)	5 (11%)	1 (2%)	1 (2%)	3.7 Easy (*N* = 47)
How easy or difficult is it to tell whether the device is functioning properly during treatment?		16 (34%)	20 (43%)	11 (23%)	0 (0%)	0 (0%)	0 (0%)	4.1 Easy (*N* = 47)
If the CPAP device malfunctions, how easy or difficult is it to identify and fix the problem?		13 (28%)	19 (40%)	15 (32%)	0 (0%)	0 (0%)	0 (0%)	4.0 Easy (*N* = 47)
How easy or difficult is it to clean and reprocess this CPAP device and its components?		15 (32%)	18 (38%)	11 (23%)	3 (6%)	0 (0%)	0 (0%)	4.0 Easy (*N* = 47)

How prepared do you feel after receiving training to use this CPAP?	Very prepared (5) Prepared (4) Neutral (3) Unprepared (2) Very unprepared (1)	16 (34%)	20 (43%)	9 (19%)	1 (2%)	0 (0%)	0 (0%)	4.1 Prepared (*N* = 46)

Overall, how satisfied are you with this CPAP device?	Very satisfied (5) Satisfied (4) Neutral (3) Unsatisfied (2) Very unsatisfied (1)	28 (60%)	15 (32%)	4 (9%)	0 (0%)	0 (0%)	0 (0%)	4.5 Very satisfied (*N* = 47)

How likely are you to recommend this CPAP system to other healthcare workers and facilities?	Very likely (5) Likely (4) Neutral (3) Unlikely (2) Very unlikely (1)	38 (81%)	8 (17%)	1 (2%)	0 (0%)	0 (0%)	0 (0%)	4.8 Very likely (*N* = 47)

*Note:* Percentages may not sum to 100 due to rounding.

**Table 4:** Healthcare worker open-response quotes.

**Theme**	**Participant**	**Comments**
Vayu bCPAP systems are easy to assemble	Participant 1	“For me, Vayu is easy to set up”
	Participant 2	“I recommend the Vayu bCPAP [more] than other types of CPAP. It is easier to set up”
	Participant 8	“Vayu CPAP… [was] easier to set up in emergency cases”

Vayu bCPAP systems are easy to operate	Participant 10	“I would recommend the Vayu CPAP since it is very handy and easy to use”
	Participant 32	“I would recommend Vayu, especially in district hospitals, as it is easy to understand”
	Participant 23	“Vayu --- easy to set up for the patient”

It is straightforward to identify whether the device is malfunctioning	Participant 16	“With Vayu CPAP, it is easier to tell if something is not functioning properly”
	Participant 34	“It is easy to determine the problem in the device with Vayu CPAP”
	Participant 15	“Vayu CPAP… does not need advanced technological skills to make its function [properly]”

The Vayu bCPAP systems are difficult to assemble	Participant 18	“Though it is hard to set up, the benefit of this device is very much commendable”
	Participant 20	“It takes time to assemble the Vayu CPAP… but it works very well”

Vayu bCPAP systems can be used in resource-limited settings	Participant 10	“I would recommend the Vayu CPAP since… it can be used in resource-limited settings like the Philippines”
	Participant 43	“[I recommend] Vayu CPAP since it can be used in remote areas where there is limited equipment”

Vayu bCPAP systems are affordable and do not require electricity or medical compressed air	Participant 25	“I will recommend Vayu CPAP to other facilities because it is a low cost-effective device compared to other devices”
	Participant 19	“I recommend Vayu CPAP for facilities with limited resources because it is more affordable”
	Participant 44	“Vayu is cheaper and does not require compressed air”
	Participant 7	“[I recommend] the Vayu CPAP because it does not require electricity”

Perceived positive neonatal outcomes following the introduction of Vayu bCPAP systems	Participant 18	“Since we started using Vayu CPAP in our institution, there was a noted decrease in the chance of a patient being intubated”
	Participant 6	“I recommend Vayu CPAP because it is a low-cost device that allows delivery of oxygen flow, concentration, and pressure that can improve the outcomes of patients and lessen the patients from intubation”
	Participant 41	“It is very effective for neonates”
	Participant 24	“Vayu CPAP is very useful to all neonates. I could say that this is an essential device in the ICU, like the mechanical ventilator, wherein many patients may recover from their critical conditions. I just hope all hospitals are capable of having more devices like this.”

Improvements for training	Participant 10	“The use of the Vayu CPAP will be enormously improved and maximized if there would be comprehensive lecture and hands-on refresher training for us”

Limitations of ventilator-driven CPAP	Participant 7	“The ventilator CPAP… it requires knowledgeable personnel to set up the machine”
	Participant 17	“[The most challenging is] Ventilator CPAP, you need the help of a respiratory therapist to set up the ventilator”
	Participant 19	“Ventilator-driven CPAP is the most challenging to incorporate into healthcare settings especially into facilities with limited resources… not all healthcare facilities can afford mechanical ventilators”

Greater availability of Vayu bCPAP systems in the ITRMC NICU	Participant 10	“The Vayu CPAP is readily available in an emergency setting when there is no mechanical ventilator”
	Participant 30	“Availability of the ventilator right away is the main problem hence Vayu CPAP is more available in our NICU setting”

## Data Availability

The data that support the findings of this study are available from the corresponding author, Dr. Paula Rauschendorf, upon reasonable request.
